# Relevance of Crop Biology for Environmental Risk Assessment of Genetically Modified Crops in Africa

**DOI:** 10.3389/fbioe.2015.00150

**Published:** 2015-10-09

**Authors:** Olalekan Akinbo, James F. Hancock, Diran Makinde

**Affiliations:** ^1^NEPAD Agency African Biosafety Network of Expertise (ABNE), Kampala, Uganda; ^2^Department of Horticulture, Michigan State University, East Lansing, MI, USA; ^3^NEPAD Agency African Biosafety Network of Expertise (ABNE), Ouagadougou, Burkina Faso

**Keywords:** African crops, risk assessment, crop biology, biotech crops

## Abstract

Knowledge about the crop biology of economic crops in Africa is needed for regulators to accurately review dossiers and conduct comprehensive environmental risk assessments (ERAs). This information allows regulators to decide whether biotech crops present a risk to biodiversity, since crossing between domesticated crops and their wild relatives could affect the adaptations of the wild species. The criteria that should be used in the evaluation of African crops for ERA include growth habit, center of origin, center of genetic diversity, proximity of wild relatives, inter-fertility, mode of pollen dispersal, length of pollen viability, mating system, invasiveness, weediness, mode of propagation, mode of seed dispersal, and length of seed dormancy. In this paper, we discuss the crops being genetic engineered in Africa and describe the crop biology of those with native relatives.

## Introduction

Active farming activities by humans began as far back as 13,000 years ago and the ancient hunter-gatherer strategy is no longer common for daily sustenance (Giguet-Covex et al., 2014). The seed and fruit structures that evolved in the angiosperms (179–158 Million years) ultimately attracted humans to domesticate them for food. The development of the carpel in angiosperms led to high levels of genetic diversity in species through natural selection (Whitehouse, 1950; De Nettancourt, 1977). Other factors, such as genetic drift, also contributed to levels of diversity in plant species (Saccheri and Hanski, 2006). The dicotyledons provided the highest number of crops, but most of the world feeds on a few monocotyledonous grains.

**Figure 1:**
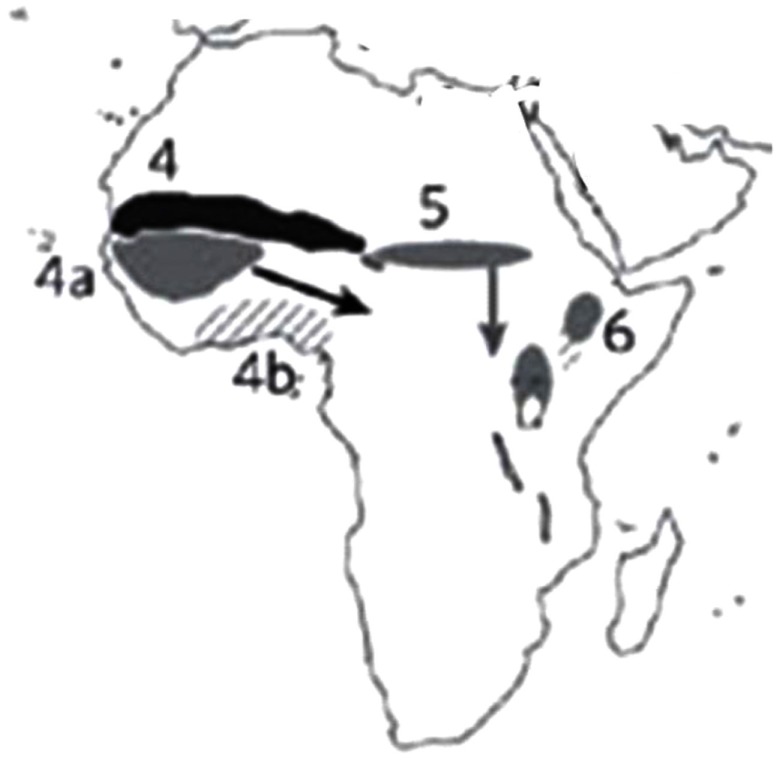
**Centers of plant domestication in Africa**. Solid-shaded areas and hatched areas indicate regions of important seed and vegetative crops domestication: 4 – West African Sub-Sahara, 4a – West African savanna and woodlands, 4b – West African rainforests, 5 – East Sudanic Africa, and 6 – East African uplands and lowland vegeculture. (Source: Purugganan and Fuller, 2009).

According to Vavilov (1926, 1949–1950), there were eight centers of domestication. Harlan (1967) identified, three relatively small “centers of origin,” but suggested three additional, larger non-focused areas called “non-centers.” In the last 20 years, additional archeological data have fine-tuned these lists (Figure 1). In the West African Sub-Sahara, pearl millet (*Pennisetum glaucum*) was domesticated. The East African uplands were the origin of finger millet (*Eleusine coracana*) and the East African lowlands yielded the yam (*Dioscorea cayenensis*). Cowpea (*Vigna unguiculata*) and African rice (*Oryza glaberrima*) were domesticated in the West African savanna and woodlands. From the West African rainforests came the yam (*Dioscorea rotundata*) (Hancock, 2012).

The adoption of modern biotechnology derived crops in Africa has been slow, as most farms are less than a hectare and support a single family with minimal resources. In addition, most farms rely on rain-fed cropping systems, with low input farm supplies, and unstructured land-tenure systems. However, increasing agricultural productivity will be critical in overcoming low yield productivities. Crop yields from African farms have fallen well below global averages and reducing the yield gap would increase farmers’ incomes and have beneficial impacts on hunger and poverty.

Biotechnology offers important opportunities to enhance yields by helping overcome the challenges imposed by diseases, drought, and relative incompatibility of species (Bailey et al., 2014). The global area of biotech crops has increased over the last nineteen years to 448 million hectares (James, 2015). The main traits incorporated into biotech crops so far commercialized convey tolerance to specific herbicides, and specific resistance to insect pests (Barfoot and Brookes, 2014). Other traits in the pipeline include vitamin and micronutrient fortification, disease resistance, and drought tolerant traits.

Twenty-eight countries are currently growing biotech crops, of which three are in Africa (Burkina Faso, South Africa, and Sudan) covering a total of 3.3 million hectares in 2014 (James, 2015). Of the total global surface of biotech crops, Bt cotton was planted on 25.1 million hectares, Bt maize was planted on 55.2 million hectares in 2014 (James, 2015). South African farmers are cultivating Bt cotton and maize on a total acreage of 3.3 million acres.

An important barrier to the broader use of biotech crops in Africa is the regulatory capacity available in individual countries to evaluate the environmental biosafety of these crops. The objective of this review is to provide regulators with the information on the biology of African crops that is needed to make accurate science-based, regulatory decisions. We also briefly review the ongoing biotechnology research being conducted in Africa.

## Crop Biology Information for the Regulators in Africa

One of the main components of the Cartagena Protocol on Biosafety is to ensure “an adequate level of protection in the field for safe transfer, handling, and use of living modified organisms resulting from modern biotechnology that may have adverse effects on the conservation and sustainable use of biological diversity, taking also into account risks to human health, and specifically focusing on trans-boundary movements.” This approach is governed by science-based risk assessment and risk management (Craig et al., 2008). Risk assessments are based on information provided by the developer, including molecular characterization, protein expression, protein toxicity and allergenicity, compositional and phenotypic analysis, types of pollination (cross-pollination vs. self-pollination), weediness of the crop, and potential routes of gene escape (vegetative, seeds, and/or pollen).

Among the foremost concerns associated with the release of biotechnology crops in Africa is that they will have a negative impact on the rich biodiversity found across the continent. Africa is a center of origin and diversity for cowpea, millet, rice, sorghum, and yam and a center of diversity for banana, cassava, potatoes, rice, and sweet potatoes. The introduction of biotech crops into centers of origin has been of particular concern, because the wild species are an untapped reservoir of genetic diversity for potential crop improvement which must be preserved (Gepts and Papa, 2003).

For gene exchange to occur, the cultivar and wild relatives must be within pollen/seed dispersal range, be able to produce viable and fertile hybrids, and overlap in flowering time (Gepts and Papa, 2003). According to Hancock et al. (1996) and Ellstrand et al. (1999), 12 out of 13 important crops have formed hybrids with their wild progenitors somewhere in the world. Although the possible effects of biotech crops on biodiversity has received the most attention, there are no compelling arguments to suggest that biotech crops are any greater a threat than conventionally bred crops (Dale et al., 2002; Lu et al., 2010). In fact, the genes associated with domestication often make crops less adapted to the natural environment and they have limited fitness, reducing their possible impact.

## Biotech Research in Africa

**Figure 2:**
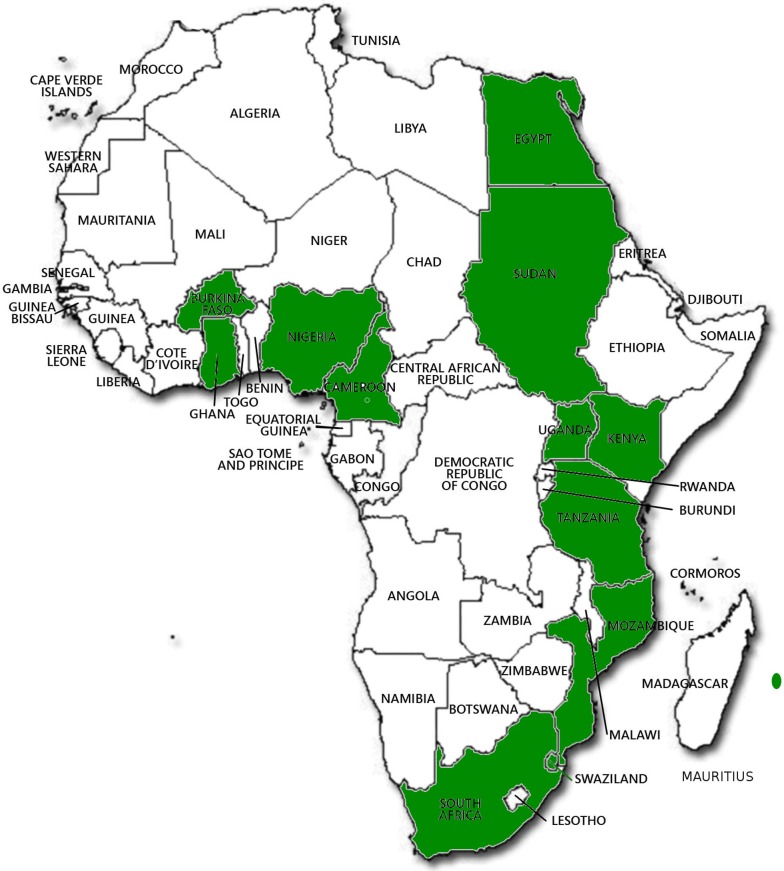
**African countries where Biotech crops have been grown and are being tested in confined field trials**. (Picture ABNE 2015 (unpublished): Ladji Sidibe of ABNE).

There are many biotech research activities going in Africa in the laboratory and field at a number of national research institutes including Burkina Faso, Cameroon, Egypt, Ghana, Kenya, Malawi, Mauritius, Mozambique, Nigeria, South Africa, Sudan, Swaziland, Tanzania, and Uganda (Table 1). The crops being engineered are banana, cassava, cotton, cowpea, maize, rice, sorghum, and sweet potato (Table 1). Confined field trials of biotech crops are being conducted in 13 countries (Figure 2).

**Table 1:** **Summary of biotech crop research being conducted in Africa**.

S/No.	Crops	Centre of origin	Traits
1	Banana	Southeast Asia	Bacterial wilt resistance, parasitic nematode and weevil resistance, bio-fortification with iron and vitamin A
2	Cassava	North-Eastern Brazil	Mosaic resistance, brown streak resistance, bio-fortification with iron, protein, vitamin A
3	Cowpea	West and Northeast Africa	Pod borer resistance
4	Maize	Mexico and Guatemala	Stem borer resistance, drought tolerance
5	Rice	Niger delta of Nigeria	Water and nitrogen use efficiency, salt tolerance
6	Sorghum	Ethiopia – Sudan region	Bio-fortification with iron and zinc
7	Sweet potato	Central/South America	Weevil resistance

*Sources – The Forum for Agricultural Research in Africa (FARA) (2011) and Namuddu and Grumet (2013)*.

## Crops of African Origin and Their Biology

Three of the crops commonly grown in Africa, banana (*Musa acuminata*), maize (*Zea mays*), and sweet potato (*Ipomoea batatas*) do not have compatible relatives in Africa, and therefore cannot transfer their genes to wild relatives through hybridization. Banana is Southeast Asia in origin and is sterile. Maize came from Central America and sweet potato from South America.

Cowpea *Vigna* is a large tropical genus with the majority of its species being found in Africa (West and Northeast). All the species have 22 chromosomes and little cytogenetic divergence. The wild progenitors are subdivided into five groups on the basis of seed and pod characteristics – *V. unguiculata* (Seed crop), *Biflora* (fodder), *Sesquipedalis* (green pod vegetable), *Textilis* (peduncle fibers), and *Melanophthalmus* (seed crop). *V. unguiculata* ssp. *unguiculata* var *unguiculata* is the cultivated form (Pasquet, 1998). DNA evidence from domesticated clones and some wild progenitors suggest Africa as the origin of cowpea spp. (West and Northeast) (Vaillancourt and Weeden, 1992; Coulibaly et al., 2002). Nigeria and Niger are the leading producers of cowpea in Africa.

Cowpea is propagated by seed and is predominantly a self-pollinated plant with the level of outcrossing being <10%. Although cowpea cultivars can grow rapidly and shade other plants out, they rarely become dominant in natural environments (Akinbo, 2013). They are insect pollinated by primarily bees and wasps. The pollen of cowpea remains viable for up to 12 h. Seeds of wild plants are dispersed from the mother plant relatively short distances. Wild-derived seeds have a thick seed coat and germinate erratically.

African rice, *O. glaberrima* was first domesticated in Africa. Its ancestors were diploid (2*n* = 24) and its cultivation probably began in the Niger Delta of Nigeria and spread across tropical East Africa (Chang, 1975). The crop and wild relatives are completely inter-fertile; however, rice is predominantly self-pollinated and it is not invasive in natural ecosystem. Clegg et al. (1993) did uncover a low, but finite amount of outcrossing where wild or weedy rice are closely associated with crop rice production. Rice pollen is dispersed by insects and wind, and remains viable for only 5–15 min. The seed do not have a dormancy period and are dispersed by shattering.

Yams are in the subspecies Dioscorea in the family *Dioscoreaceae*. The genus *Dioscorea* contains about 600 species but only 10 species are edible (Akoroda, 1983). The most important cultivated species are *D. cayanensis*, *D. rotundata*, *D. alata*, *D. dumetorum*, and *D. bulbifera* (Dansi et al., 2001; Arnau et al., 2010; Obidiegwu et al., 2010). The base chromosome number of *Dioscorea* is *x* = 20, likely making it a polyploid (Hochu et al., 2006; Bousalem et al., 2010). The cultivated *D. rotundata* and *D. cayenesis*, *D. alata*, *D. dumentorum* are grown in the “yam belt,” i.e., from Central Côte d’Ivoires through the mountains of Cameroon (Lagemann, 1977; Asiedu et al., 1992). *D. rotundata* was domesticated in West Africa (Dumont et al., 2005). The likely wild ancestors of *D. rotundata* in the savannah area are *D. abyssinica* Hochst ex Kunth and in humid forests *D. praehensilis* Benth.

Sadik and Okereke (1975) reported that unisexuality and dioecism is common in yam, although, complete flowers have been observed in some genotypes. The pollen grains are sticky and cannot be dispersed by wind; pollen grain viability appears to be low (12%). Fruit and seed set are also very low. Yam can be persistent in the environment but is non-invasive (Hancock, 2015). Its pollen is dispersed by insects and remains viable for very short periods. Seed dispersal is through winged seeds.

Millet is another crop of Africa origin and is represented by a number of different genera, including *Pennisetum americanum* (L.) Schum and *E. coracana*. Pearl millet (2*n* = 2*x* = 14) belongs to a highly heterozygous group. Finger millet (2*n* = 4*x* = 36) has both diploid and allotetraploid races. Oumar et al. (2008) suggested that Pearl millet cultivation originated in tropical, West Africa. The diploid finger millet originated in tropics and subtropics, and the tetraploid of finger millet are from eastern and southern Africa. The origin of *E. indica* ssp. *africana* indicated by molecular data was probably Eastern Africa (Salimath et al., 1995). Millets are outcrossing (more than 85%); both Finger millet and Pearl millet are not invasive in natural environments but can be weedy in agricultural fields (Ellstrand et al., 2010).

**Figure 3:**
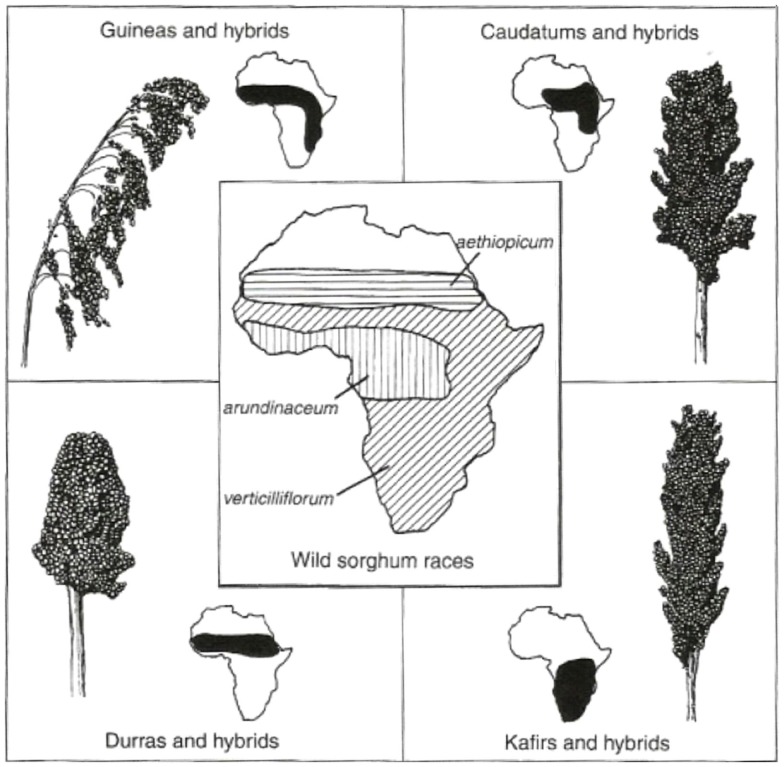
**Distribution of wild and cultivated races of sorghum**. [Used with permission from Hancock (2012)©].

Last but not least in the group of crops known to be of Africa origin is Sorghum (Figure 3). Wild Sorghum has wide morphological variability and a complex taxonomy, represented by over 70 species (Doggett, 1988). There are three main species, namely, *Sorghum bicolor* ssp. a*rundinaceum*, *Sorghum halepense*, and *Sorghum propinguum*. *S. bicolor* ssp. *arundinaceum* is an allotetraploid (2*n* = 2*x* = 20) and is found in tropical Africa. Morphological and molecular variability revealed that sorghums center of origin is in the Ethiopia–Sudan region (Perumal et al., 2007). The cultivated sorghum in Africa are represented by four subspecies *Bicolor* is planted across Savannah Africa, *Caudatum* in Central Sudan, Guinea in Eastern and Western Africa, *Durra* in Ethiopia and the Nile Valley, *Kafir* in Southern Africa. These have been grouped by molecular fingerprints (AFLP and SSR data) (Perumal et al., 2007).

Sorghum is predominantly self-pollinated with limited cross-pollination between cultivated and wild relatives; sorghum is highly domesticated and cultivars generally do poorly in the wild; however, wild genotypes and crop/wild hybrids can be invasive and weedy (Akinbo, 2013). Pollen remains viable for up to 20 h. Seed are dispersed by wind and animals and can remain viable in the soil for years.

## Challenges with Respect to ERA

Although the Organization for Economic Co-operation and Development (OECD) has produced consensus documents on the crop biology of the African crops rice, sorghum, potatoes, maize, wheat, cotton, and cassava, others of importance in Africa like yam, cowpea, and millet are yet to be developed. One of the challenges of environmental risk assessment (ERA) is to build risk management strategies that are firmly based on the reproductive biology of indigenous crops (Renn, 1998). The complexity of ecological systems can present considerable challenges as potential impacts may vary spatially and temporally. Unfortunately, for many crops of African origin, little baseline information is available on the distribution of the crop and its wild relatives.

## Way Forward

The promise of increased productivity using biotechnological techniques has been documented in the literature world-wide, and directly in Burkina Faso, South Africa, and Sudan. Over 43 African countries have signed and ratified the Cartagena Protocol on Biosafety, but only 12 have put in place a robust and workable regulatory system. Until a country has a functional regulatory system, the benefits of biotechnology cannot be utilized. A competent authority (National Biosafety Agency) must be identified within the various governmental ministries, which is empowered by the Biosafety Law to handle applications and take decisions based on the recommendation of the technical experts. The application review process in Burkina Faso is a good example of a regulatory structure that takes into account the precautionary principle laid out in the Cartagena Protocol on Biosafety.

## Conflict of Interest Statement

The authors declare that the research was conducted in the absence of any commercial or financial relationships that could be construed as a potential conflict of interest.
